# Stable disease, diagnosis and dose are prognostic factors for survival after interleukin-2 immunotherapy

**DOI:** 10.1186/2051-1426-3-S2-P138

**Published:** 2015-11-04

**Authors:** Nathan H Kwan, Brittany A Ockenfels, Charmi Patel, Gerald P Miletello, Paula R Brantley

**Affiliations:** 1 Tulane University School of Medicine, New Orleans, LA, USA; 2 Baton Rouge General Internal Medicine Residency Program, Baton Rouge, LA, USA; 3 Hematology/Oncology Clinic, Baton Rouge, LA, Baton Rouge, LA, USA; 4 Baton Rouge General Medical Center Graduate Medical Education, Baton Rouge, LA, USA

## Background

Recent studies have reported that the clinical benefit of interleukin-2 (IL-2) immunotherapy for metastatic renal cell carcinoma (mRCC) and melanoma (mM) may be underestimated by typically reported outcomes, instead suggesting that progression-free survival or “disease control rate” (DCR) may provide a more meaningful prognostic indicator [1]. Therefore, we examined progression-free survival to assess the clinical benefit of IL-2.

## Methods

A total of 78 patients in Baton Rouge, Louisiana enrolled in the PROCLAIM registry from 2011 to 2015 were reviewed, and 58 patients had complete survival and response data. Patients received IL-2 600,000 IU/kg intravenous boluses every 8 hours for up to 14 doses (one cycle), up to 4 cycles. Using the Response Evaluation Criteria in Solid Tumors v1.1, treatment response was assessed as complete response (CR), partial response (PR), stable disease (SD), or progressive disease (PD) after cycle 2, cycle 4, and at each follow-up. Univariate analysis of dichotomous or categorical predictors of survival was performed using the Kaplan-Meier method, and differences were compared by the log-rank test using Stata 12. Landmark analysis was conducted at 6-month, 1-year, 2-year, and 3-year time points. A two-tailed significance level of 0.05 was used to construct a Cox proportional hazards regression model to obtain hazard ratios (HR) for predictors of survival.

## Results

**Figure 1:**
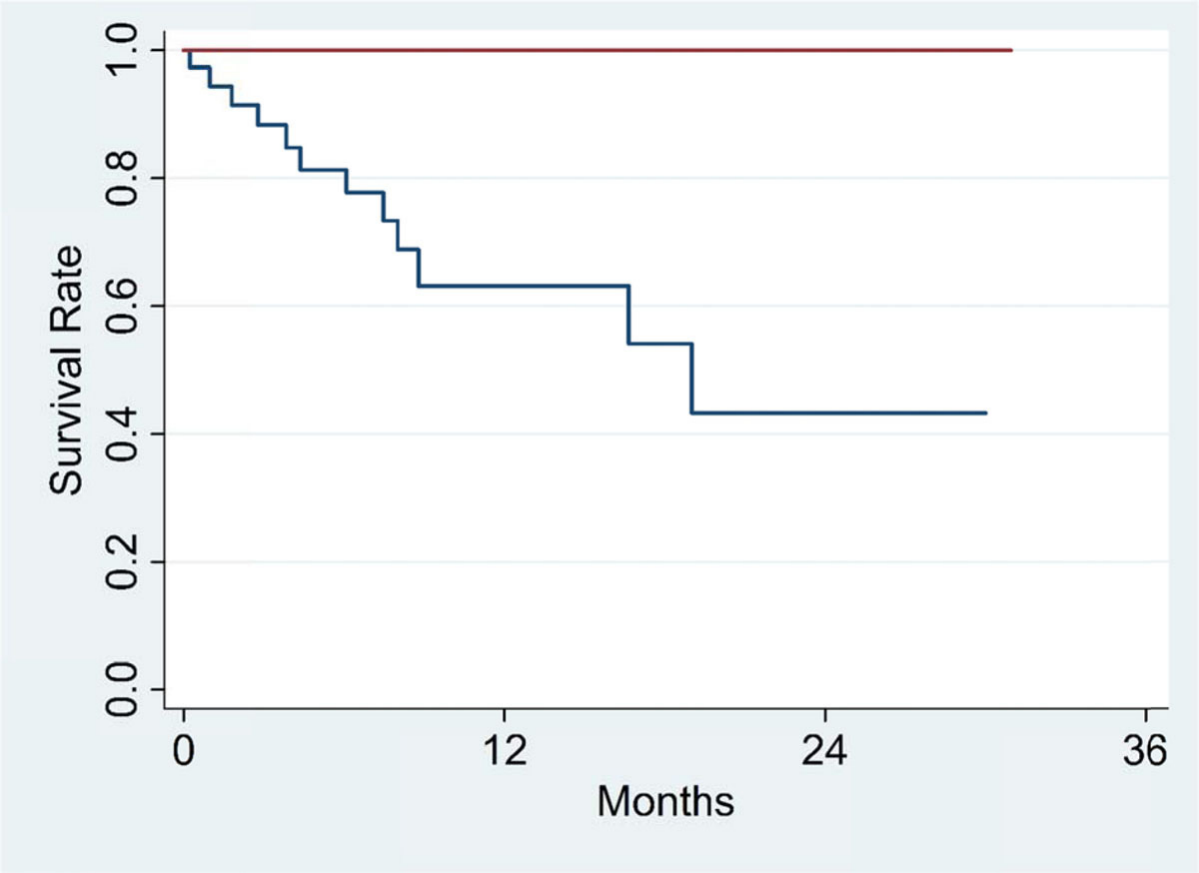


**Figure 2:**
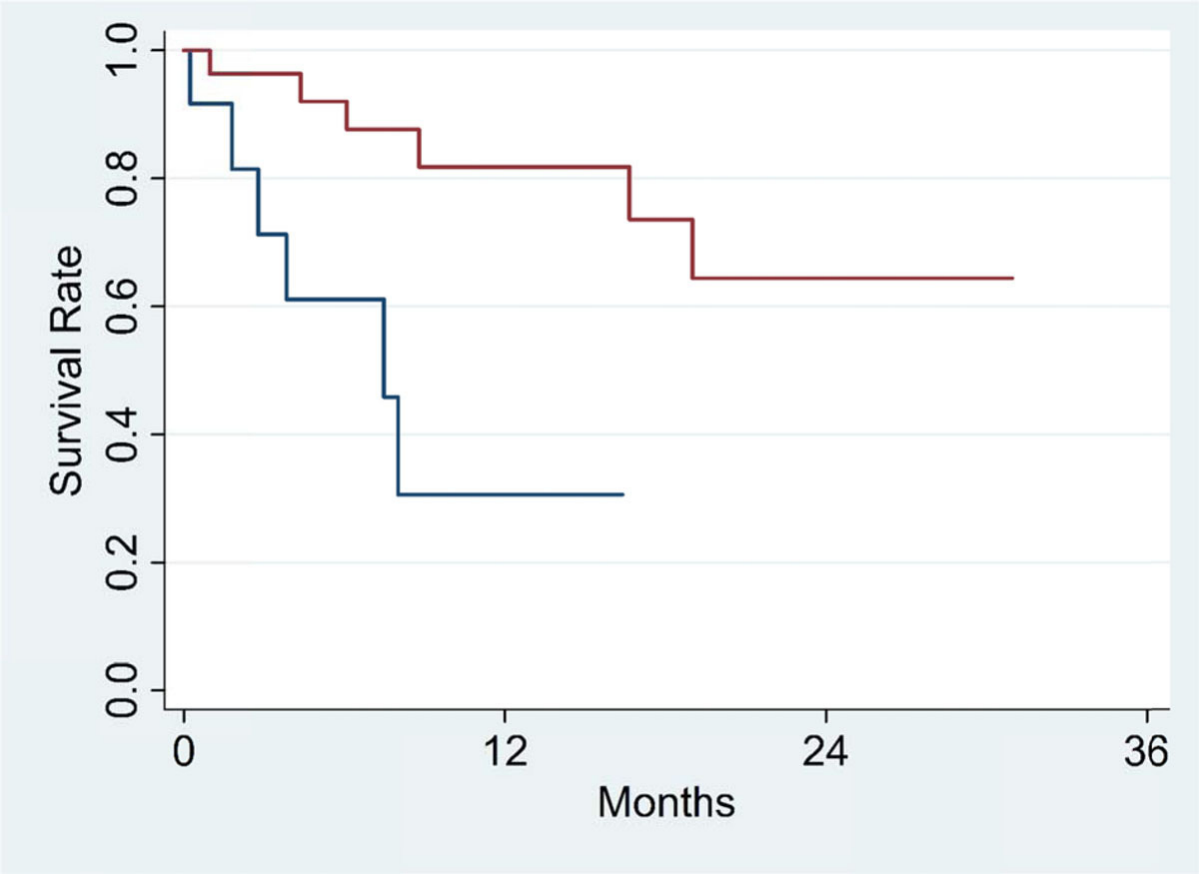


To control for guarantee-time bias, the 6-month landmark from start of treatment was chosen, at which time all patients had completed all cycles of IL-2. From the 6-month landmark, CR, PR, and SD did not reach median survival while PD had a 7.5 month median survival (p=0.014). Differences in survival between DCR (Figure 1, red line) and PD are significant (p=0.002) while differences between ORR (Figure 2, red line) and non-objective responders are not statistically significant (p=0.096). Number of doses (p=0.000; HR=0.891; 95% CI, 0.850 to 0.935) and primary diagnosis of mRCC (p=0.004; HR=0.248; 95% CI, 0.095 to 0.648) were significant covariates associated with survival in the final Cox model.

## Conclusions

Although our findings may have limited generalizability, in this sample stable disease, a primary diagnosis of mRCC, and the total number of IL-2 doses were significantly associated with prolonged survival from the 6-month landmark. In addition to ORR, progression-free survival should be reported to reflect the clinical benefit of IL-2 therapy.

## References

[B1] Hughes T Klairmont M Broucek J Iodice G Basu S Kaufman HL The prognostic significance of stable disease following high-dose interleukin-2 (IL-2) treatment in patients with metastatic melanoma and renal cell carcinoma Cancer Immunol Immunother 2015 64 4 459 465 25603775 10.1007/s00262-014-1652-6 PMC4448124

